# Holding it together: ABCG43 regulates root-soil interactions via root exudates

**DOI:** 10.1093/plphys/kiaf229

**Published:** 2025-06-05

**Authors:** Avilash Singh Yadav

**Affiliations:** Assistant Features Editor, Plant Physiology, American Society of Plant Biology; Weill Institute for Cell and Molecular Biology and Section of Plant Biology, School of Integrative Plant Sciences, Cornell University, Ithaca, NY 14853, USA

“*There's nothing under the ground that's worth more than the little layer of topsoil sitting on top of it.”—Wendell Berry*.

It is common knowledge that the nutrient-rich topsoil is essential for plant growth. However, soil erosion depletes the fertile soil layer, which impairs the ability of soil to retain water and nutrients (Montgomery 2007). Soil erosion is a major threat to food security, as it can render farmlands unsuitable for cultivation. While improvements in agricultural practices to alleviate soil erosion are underway, emerging approaches involve leveraging plant biology to develop crops that can bind firmly to the soil (Sadikiel Mmbando and Ngongolo 2024).

It is well known that plant roots physically hold the soil together; however, what traits determine how strongly the roots adhere to the surrounding soil is unclear. Structural traits such as the length of lateral roots and the number and density of root hairs contribute to how firmly roots bind to the soil (Bailey et al. 2002). However, root architecture is only a part of the story. Roots also secrete organic compounds called root exudates, which can affect soil-binding properties and influence the root-soil cohesion (Badri and Vivanco 2009; Akhtar et al. 2018). Plants release root exudates through both passive and active mechanisms. Passive processes involve shedding of the root cap cells (sloughing), diffusion, and secretion, whereas active mechanisms involve 2 major families of transporters: ATP-Binding Cassette (ABC) and Multidrug And Toxic Compound Extrusion (MATE) (Miao and Liu 2010).

The ABC family of transporters is well conserved in plants, wherein the ABCG subfamily is the largest and functionally the most diverse subfamily of membrane transporters (Dhara and Raichaudhuri 2021). Although ABCG proteins are primarily involved in environmental stress responses, they are also involved in modifying root–soil interactions. For instance, in *Arabidopsis thaliana*, ABCG30 influences the composition of root exudates to enhance root-gel adhesion (Badri et al. 2009). Similarly, ABCG43, initially shown to confer cadmium tolerance in rice, has subsequently been shown to influence root–substrate interactions in Arabidopsis (Oda et al. 2011; Eldridge et al. 2021). However, the precise mechanism through which ABCG43 mediates root–substrate interactions remain unclear.

Recently, in *Plant Physiology* (Eldridge et al. 2025), the authors show that ABCG43 modulates the composition of root exudates to regulate root–substrate cohesion in Arabidopsis. Based on phylogenetic analysis, the authors found that *ABCG43* belongs to a plant-specific subfamily that includes several closely related genes. Interestingly, several major crop species, including rice, maize, soybean, tomato, and potato, have at least 1 ortholog of Arabidopsis *ABCG43* with a high sequence similarity (E-value < 3.81e-148). The conserved nature of *ABCG43* sequences indicates that *ABCG43* is a promising target for improving root–soil interactions in crops.

Based on RT-PCR analysis, the authors found that *ABCG43* is predominantly expressed in the root tissue. To then detect the subcellular localization of *ABCG43* in the roots, the authors generated transgenic lines in the *abcg43* mutant backgrounds expressing *ABCG43-GFP* under the constitutive *UBQ10* promoter. Confocal imaging confirmed that *ABCG43-GFP* co-localizes with the plasma membrane in the root epidermal cells, consistent with ABCG43 being a membrane transporter (Figure). Interestingly, *ABCG43-GFP* was also detected in small spots (puncta) inside the cells of the root tip, suggesting that ABCG43 may be directly trafficked to the membrane.

**Figure.**
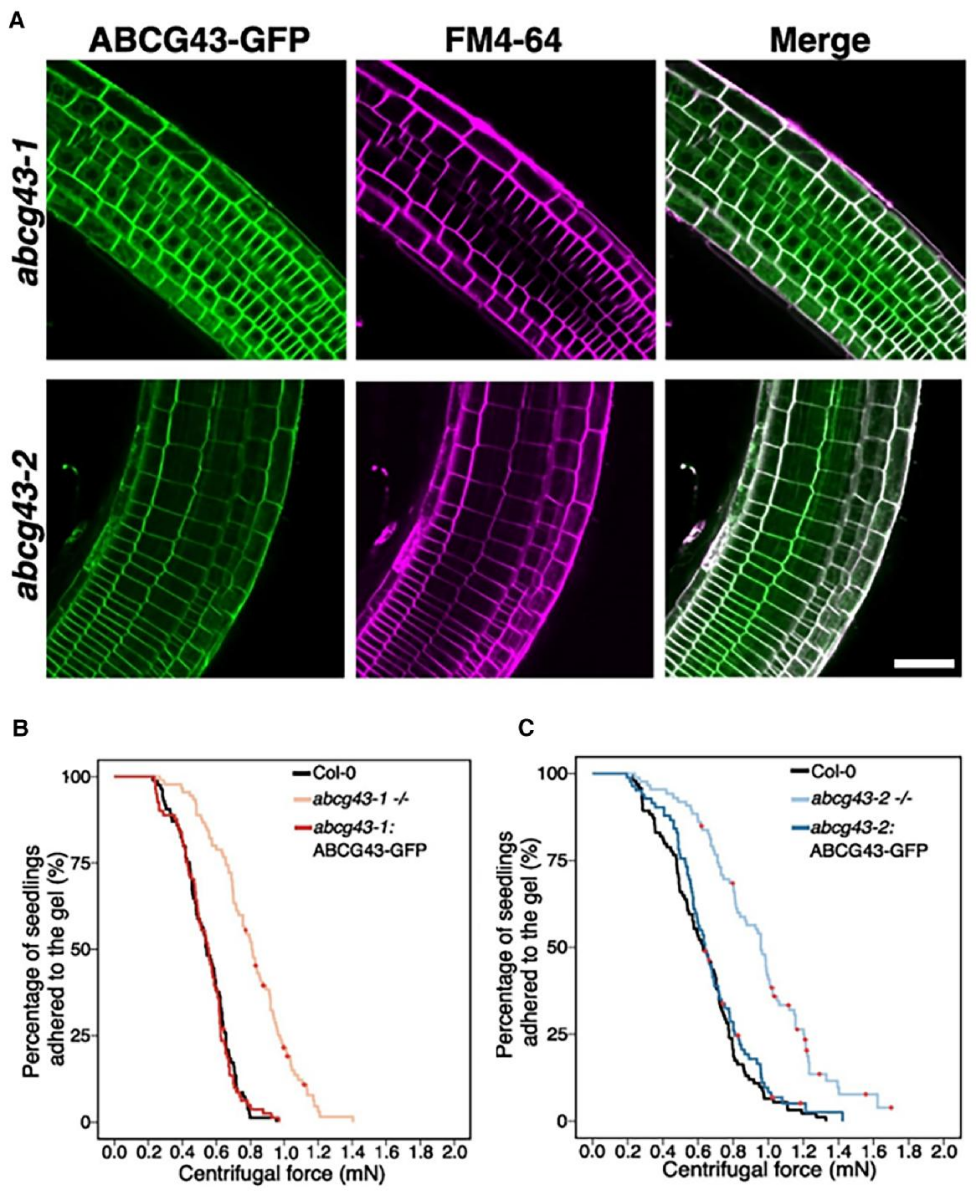
Membrane transport protein ABCG43 modulates root–substrate adhesion. **A)** Confocal images of the root epidermal cells expressing *pUBQ10::ABCG43-GFP* in 2 independent *abcg43* mutant alleles (*abcg43-1* [top] and *abcg43-2* [bottom]). From left to right, the panels show GFP fluorescence, plasma membrane dye FM4-64, and the merged image (showing colocalization), respectively. Scale bar, 50 *µ*m. **B**, **C)** Plots show the results of root–substrate adhesion assay for **B)**  *abcg43-1* and **C)**  *abcg43-2*. The percentage of seedlings that remain attached to the gel substate is plotted against the centrifugal force applied. Both *abcg43-1* (b: light red) and *abcg43-2* (c: light blue) show increased adhesion compared to wild-type Col-0 (black). Root-gel adhesion for the transgenic lines constitutively expressing *ABCG43-GFP* in *abcg43-1* (b: red) and *abcg43-2* (c: blue) is indistinguishable from Col-0. Adapted from (Eldridge et al. 2025).

The authors then asked whether ABCG43 influences root adhesion in a dose-dependent manner. Toward this, they used lines expressing varying levels of ABCG43 to perform centrifuge-based root adhesion assays, which quantify the force required to detach seedlings from a gel substrate. The authors found that the homozygous *abcg43* mutants exhibit significantly stronger root-gel adhesion compared to the wild type (Col-0) (Figure), while the heterozygous mutants exhibit an intermediate phenotype. Thus, loss of *ABCG43* increases cohesion between roots and substrate in a dose-dependent manner. Moreover, no significant differences were observed in the length and density of root hairs across genotypes, thus ruling out the possibility of morphological differences underlying the differences in root-gel adhesion.

Building on these insights, the authors grew the lines expressing varying levels of *ABCG43* in a complex growth medium until the late vegetative stages. A tensile testing apparatus was then used to uproot the plants in a controlled manner and quantify the amount of growth medium adhering to the roots. Interestingly, *abcg43* mutants retained 2.2 to 2.6 times more growth medium per unit root length relative to Col-0. The observed differences were specifically due to the loss of *ABCG43*, since *abcg43* mutants complemented with functional *ABCG43* showed substrate retention levels comparable to Col-0. Overall, these findings demonstrate that ABCG43 affects root–substrate adhesion through mechanisms independent of root morphology.

Finally, to test whether altered exudate composition underlies the increased root–substrate adhesion in *abcg43* mutants, root exudates were collected from Col-0 and *abcg43* mutants to perform metabolomic profiling using liquid chromatography-mass spectrometry. The *abcg43* mutants exhibit increased levels of several dipeptides, nucleoside, and organic acids, suggesting a clear difference in exudate compositions. The authors also confirmed the functional relevance of altered exudate compositions by performing substrate binding assays. As expected, the exudates from *abcg43* mutants were found to bind 2.43 to 3.08 times more growth medium and 1.71 to 2.20 times more soil relative to Col-0, which is consistent with the root adhesion assays.

Taken together, the authors (Eldridge et al. 2025) demonstrate how ABCG43 influences root–substrate interactions by modulating the composition of root exudates rather than altering root morphology. This study provides insights into the molecular regulation of root–soil cohesion, which is important for mitigating soil erosion. Going forward, investigating the functional roles of *ABCG43* orthologs in crop species will be helpful to sustainable agriculture. It is also worth exploring how ABCG43-mediated exudates influence microbial communities in field conditions, which could offer strategies to engineer rhizosphere dynamics.

## Data Availability

There are no new data associated with this News & Views article.
